# A novel approach utilizing spirocyclic thiopyrimidinone compounds against herpes simplex virus with underlying antiviral mechanisms of action

**DOI:** 10.1186/s12985-025-02707-9

**Published:** 2025-04-11

**Authors:** Esraa A. Manie, Emad M. Elzayat, Sherif S. Ragab, Ayman M. Sweed, Hosny Ibrahim, Sherif Nasser, Abdou K. Allayeh

**Affiliations:** 1 Biotechnology Program, Faculty of Science, Cairo University, Dokki, Giza Egypt; 2 Department of Biotechnology, Faculty of Science, Cairo University, Dokki, Giza Egypt; 3 Photochemistry Department, Chemical Industries Research Institute, National Research Centre (NRC), 33 El-Behouth St., Dokki, Giza 12622 Egypt; 4 Department of the Chemistry of Natural and Microbial Products, Pharmaceutical and Drug Industries Institute, National Research Centre (NRC), 33 El–Behouth St., Dokki, Giza 12622 Egypt; 5 Chemistry Department, Faculty of Science, Cairo University, Dokki, Giza Egypt; 6 Department of Pharmaceutical Chemistry, Faculty of Pharmacy and Biotechnology, German University in Cairo, New Cairo City, Cairo 11835 Egypt; 7 Virology Lab, Water Pollution Research Department, Environment and Climate Change Institute, National Research Centre, Dokki, Giza 12622 Egypt

**Keywords:** Antiviral activity, Mechanism of action, Spirocyclic thiopyrimidinones, Herpes simplex virus

## Abstract

In recent decades, viral outbreaks have significantly threatened global health, with herpes simplex virus type 2 (HSV-2) being one of the most prevalent infections. This study evaluated novel spiropyrimidine derivatives as potential antiviral agents against HSV-2, building on previous research that examined spirocyclic thiopyrimidinone derivatives against human coronavirus 229E (hCoV-229E). Among the eleven synthesized compounds, spiropyrimidinone derivative **3** demonstrated promising antiviral activity, with a selectivity index of 11.2. The drug mechanism of infection studies indicated that compound **3** primarily inhibits HSV-2 at the viral adsorption stage, achieving approximately 83% inhibition and reducing viral multiplication by 34%. Its efficacy is linked to its diketone moiety, which is known for its ability to enhance antiviral effects. Furthermore, the effect of compound 3 on viral inhibition is reflected in the level of caspase-3 protein expression, revealing that the apoptotic pathway is modulated. Docking studies revealed multiple interactions with herpes virus entry mediator (HVEM), indicating its potential as an entry inhibitor. These findings confirm that compound **3** could be a potential candidate for further development in HSV-2 antiviral therapy.

**Graphical Abstract:**

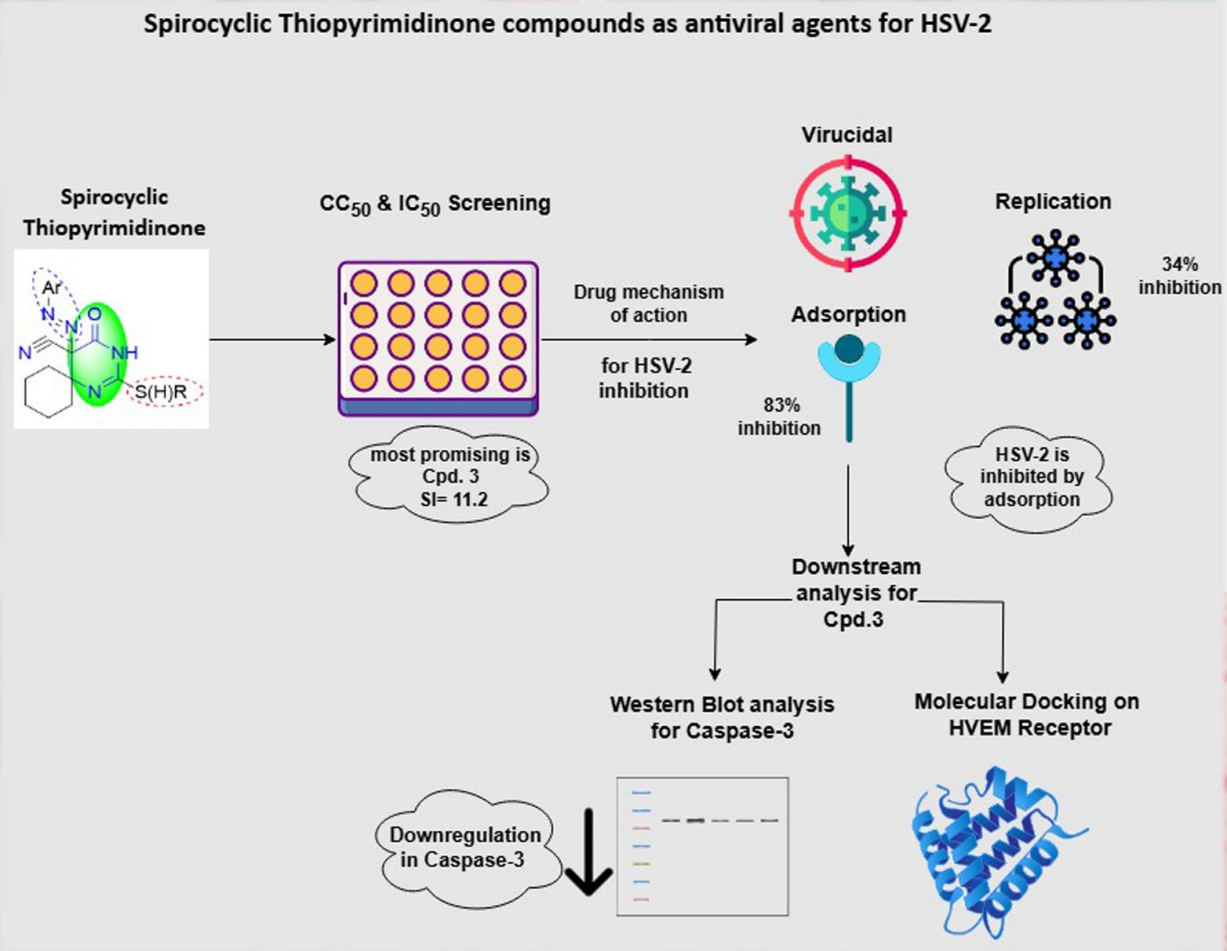

## Introduction

In the past few decades, humanity has faced numerous large-scale outbreaks of viral diseases that have posed significant threats to human life and well-being. Examples of such outbreaks include COVID-19, avian influenza, Zika virus, and Monkeypox, among other various viral infections that affect humans. Herpes simplex virus stands out as one of the most prevalent, with over two-thirds of the global population being infected [1]. The World Health Organization (WHO) reported that herpes simplex virus type 2 (HSV-2), which is transmitted primarily through sexual contact, affects approximately 500 million individuals worldwide between the ages of 15 and 49. Each year, there are approximately 20 million new cases of HSV-2, surpassing the number of new cases of other sexually transmitted diseases (STDs) or the number of sexual partners. Notably, the prevalence of HSV-2 is particularly high in sub-Saharan Africa, reaching 70%. This high prevalence in the region may contribute to the transmission and spread of HIV-1 [2].

Herpes simplex virus type 2 is a double-stranded DNA virus enveloped with a multitude of glycoproteins on its surface. These glycoproteins play crucial roles in viral propagation and are subject to cleavage by proteases, highlighting the intricate interplay between different viral components. HSV-2 belongs to the Herpesviridae family, specifically the Alphaherpesvirinae subfamily, which includes eight human viruses capable of causing chronic infections and psychological distress [3]. Following the initial infection, herpes viruses establish latency within the sensory neural ganglia, typically the sacral ganglion, in the case of HSV-2 infection [4]. HSV infections are pervasive and can manifest in various forms, ranging from localized mucocutaneous lesions to disseminated infections affecting individuals of all age groups. Neonatal herpes simplex virus disease, in particular, carries a high risk of morbidity and mortality [5]. Widespread HSV-2 infection can lead to painful blisters or ulcers that commonly affect the skin, mouth, lips, eyes, and genitals, leading to a range of diseases, such as encephalitis, meningitis, cold sores, genital herpes, and herpes stromal keratitis. Unfortunately, no vaccines are currently available for the prevention of HSV-2 infection [6].

**Fig. 1:**
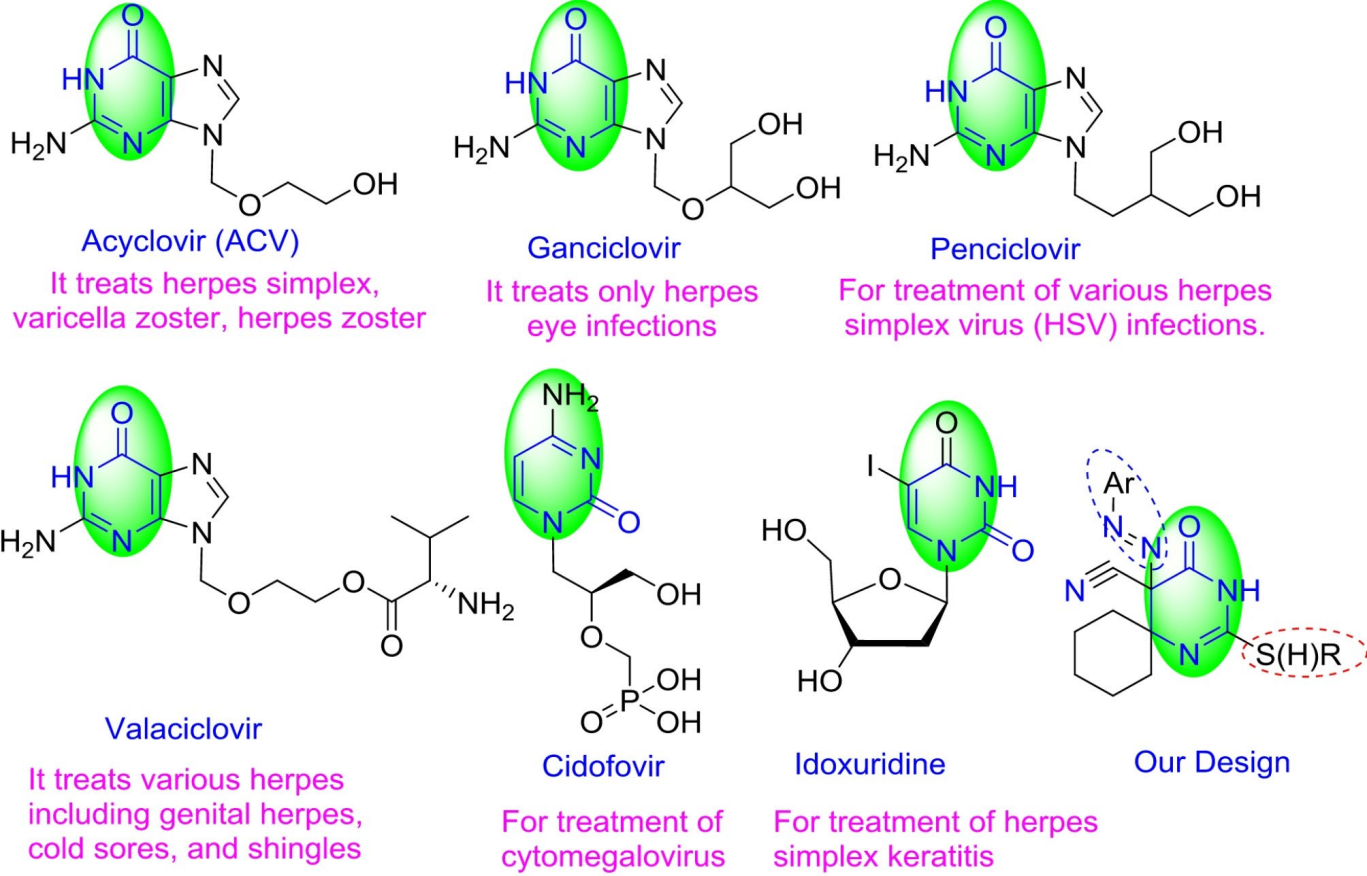
Antiherpes drugs containing pyrimidine moieties

The effectiveness of current antiviral drugs against HSV-2 is limited by their potency and the potential for adverse side effects. These drugs can be classified into two categories: nucleoside inhibitors and nonnucleoside inhibitors. Examples of nucleoside inhibitors include acyclovir, penciclovir, ganciclovir, famciclovir, and valacyclovir. Noninoside inhibitors include foscarnet, cidofovir, and docosanol [7]. However, the emergence of drug-resistant strains of the virus has highlighted the urgent need for more efficient treatment options. To address this challenge, researchers have developed a diverse range of pyrimidine-based antiherpes drugs, some of which are already approved and available on the market (Fig. 1).

Structurally, acyclovir, ganciclovir, valacyclovir, and penciclovir share a common aminopurine scaffold, specifically the imidazopyrimidine moiety, but with different substituents at the 9-position. These compounds are nucleoside analogs and are among the most commonly used drugs for HSV-2 treatment. On the other hand, cidofovir is an acyclic monophosphate nucleotide analog with an aminopyrimidinone fragment. Idoxuridine, on the other hand, is a nucleoside analog with an iodopyrimidindione moiety resulting from the modification of deoxyuridine [8]. In continuation of the efforts dedicated to the development of antiherpes drugs, we synthesized new nonnucleoside siprothiopyrimidine derivatives to investigate their antiherpes activities.

**Fig. 2:**
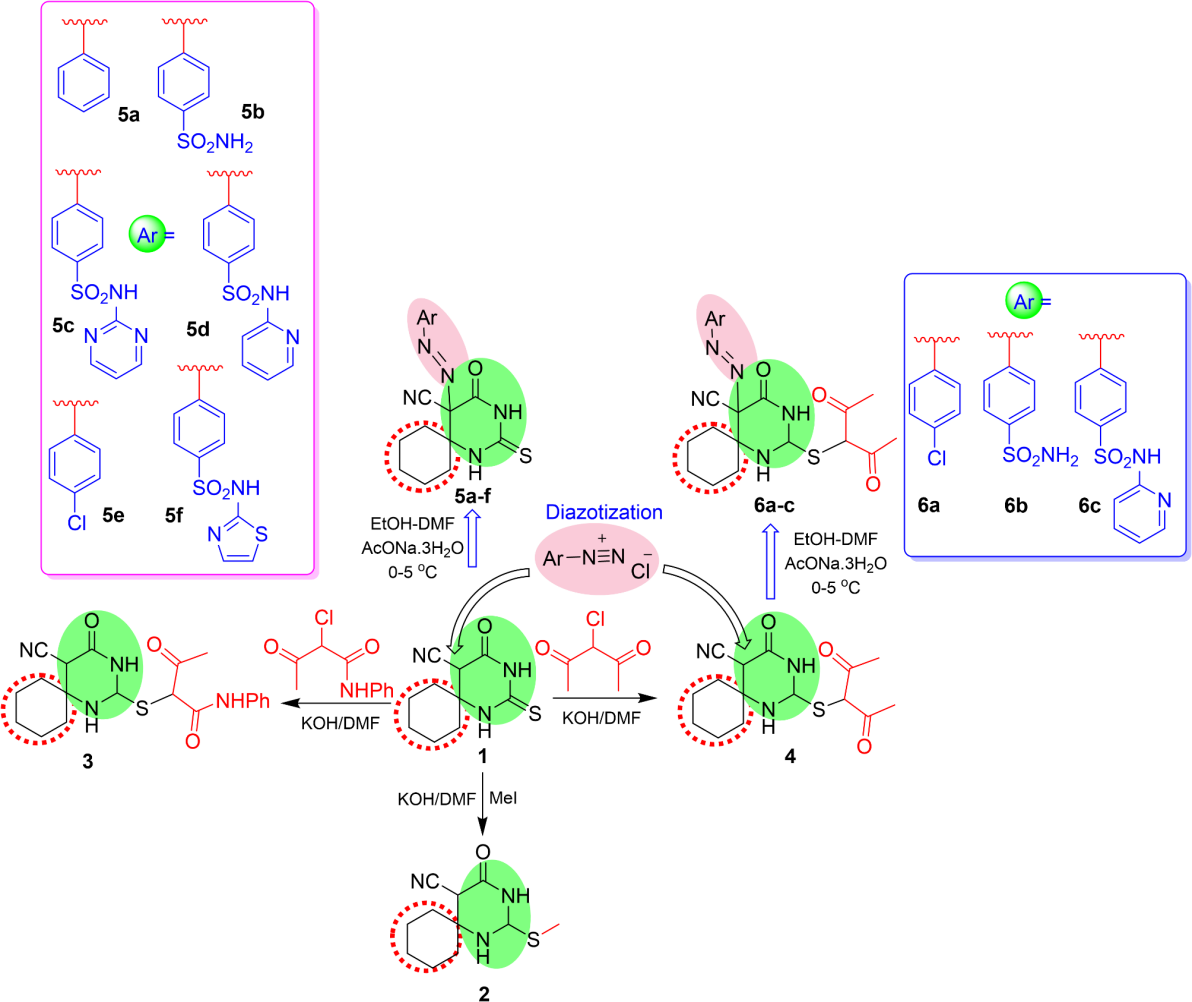
Synthesis of spirocyclic thiopyrimidinones 2–6

## Materials and methods

### Preparation of the compounds, cells and virus

All synthetic compounds were prepared according to the methods of Ragab et al. [9] and dissolved in dimethyl sulfoxide (DMSO) to prepare a 100 mg/ml stock, and adherent African green monkey kidney cells (Vero) were cultured in Dulbecco’s modified Eagle’s medium (DMEM) supplemented with 10% fetal bovine serum (FBS) and a 1% antibiotic penicillin/streptomycin mixture. The human herpes simplex virus type 2 was kindly provided by Nawah-Scientific Centre, Egypt.

Statistical analysis: The data were analyzed via GraphPad Prism analysis software version 5 for Windows (developed by GraphPad Software, Inc., USA). The data were analyzed via Student’s t-test and ANOVA (analysis of variants) via the SPSS statistical software package version 20. The data are expressed as the means and standard deviations (SDs). *p* < 0.05 was considered to indicate statistical significance.

### Screening for cytotoxicity via a crystal Violet assay

A volume of 100 µL of cell suspension containing 10^4^ cells was placed in each well of a 96-well plate. The plates containing the cells were then placed in a 5% humidified CO_2_ incubator at 37 °C for 24 h to allow the cells to form a confluent monolayer. By twofold serial dilution, each compound was diluted in 0.01 mL of medium, starting with a concentration of 1000 µg/ml. The diluted compounds were then cultured for 72 h at 37 °C in an environment with 5% CO_2_. Each concentration was tested 3 times. After the incubation period, the supernatant was discarded, and the cells were washed with 1x phosphate-buffered saline (PBS). The cells were then fixed with 100 µL of 10% formaldehyde and incubated at room temperature for 20 min. The cells were subsequently washed five times with running water. Next, 50 µL of a 0.1% solution of crystal violet dye (LOBA CHEMIE. LTD; C.I.No. 42555) was added to each well and incubated for 15 min. The cell monolayers were washed again and allowed to air dry. Finally, the crystal violet dye was dissolved by adding 200 µL of methanol, and the mixture was incubated for 20 min. The absorbance was measured via an ELISA plate reader (FLUOstar Omega Ω) at a wavelength of 570 nm [10]. Cell viability was calculated as a percentage of the average optical density of the cell controls, which was set to 100%. The 50% cytotoxic concentration (CC_50_) was calculated via mean dose‒response curves via GraphPad Prism.

### Screening the antiviral activity by MTT

In accordance with the previous section, the CC_50_ (50% cytotoxic concentration) of each compound was determined as the starting point for screening its antiviral activity. A volume of 100 µL of cell suspension containing 10^4^ cells was added to each well of a 96-well plate. The plates with the cells were then placed in a 5% humidified CO_2_ incubator at 37 °C for 24 h to allow the cells to adhere and form a monolayer. The cell monolayers were subsequently washed once with 1x phosphate-buffered saline (PBS) and infected with herpes simplex virus type 2 (HSV-2) (MOI = 1) in triplicate for 1 h. After the infection period, 100 µL of each compound’s serial dilution was added to the infected cells. A triplicate of untreated infected cells was included as a positive control (VC), and the mixture was incubated for 72 h at 37 °C in a 5% CO_2_ incubator. Following the 72-hour incubation, the supernatant medium was removed, and the cell monolayers were washed with 1x PBS. Then, 25 µL of a 0.5 mg/mL solution of MTT (3-(4,5-dimethylthiazol-2-yl)-2,5-diphenyltetrazolium bromide) obtained from Sigma‒Aldrich, Saint Louis, MO, USA, was added to each well and incubated for 2 h at 37 °C. The MTT reagent was subsequently discarded, and 200 µL of acidified isopropanol (1 N HCl in 99.9% isopropanol) was added to dissolve the formazan crystals formed by the viable cells. Finally, the color intensity was measured, and the absorbance was recorded via an ELISA plate reader. The data obtained were used to calculate the IC_50_ values (50% inhibitory concentration) via nonlinear regression analysis. This was accomplished by plotting the logarithm of the inhibitor concentration against the normalized response. The analysis was performed via GraphPad Prism 5 analysis software [11, 12].

### Identifying the Inhibition mechanisms of action

To gain a better understanding of how each compound’s antiviral activity inhibits specific steps in the viral cycle, we investigated three mechanisms: virus adsorption, virus replication, and viral fusion (virucidal). For the adsorption mechanism, we seeded 100 µl of cell suspension into 96-well cell culture plates under the previously mentioned conditions. After 24 h, the medium was discarded, and the adherent cells were washed with 1x PBS. Then, we added 100 µl of each promising compound at a pretested safe cytotoxic concentration (CC_50_) to the cells in triplicate. The plates were then incubated at 4 °C for 1 h to allow the compounds to adsorb onto the cell receptors. After incubation, the compound was removed, and the cells were washed again with 1x PBS. Next, we added 100 µl of the virus mixture (MOI = 1) to each well and incubated it for 1 h at 37 °C in a CO_2_ incubator. The virus was removed, and the cells were washed once more with 1x PBS. Finally, we added 100 µl of maintenance media containing 3% bovine serum albumin (BSA) and incubated the plates for 72 h at 37 °C in a 5% CO_2_ incubator. For the replication mechanism, we added 100 µl of diluted virus (MOI = 1) to a preseeded 96-well cell culture plate and incubated it for 1 h at 37 °C in a CO_2_ incubator. After incubation, the virus was removed, and 100 µl of each compound was added in triplicate. The plates were then incubated for 1 h at 37 °C in a CO_2_ incubator. Next, 100 µl of maintenance medium was added to each well. For the virucidal mechanism, we mixed an equal volume of the safe concentration of the pretested compound with the virus dilution (MOI = 1) and incubated the mixture at room temperature for 1 h. We subsequently prepared twofold serial dilutions of the mixture and inoculated them into the cells, which were then incubated for 1 h at 37 °C in a CO_2_ incubator. After incubation, the supernatant was removed, the cells were washed, and 100 µl of maintenance media was added. The plates were then incubated for 72 h at 37 °C in a 5% CO_2_ incubator. Following the incubation periods, we removed the medium and washed the cell monolayer with 1x phosphate-buffered saline (PBS). Next, we added 25 µL of 0.5 mg/mL MTT (3-(4,5-dimethylthiazol-2-yl)-2,5-diphenyltetrazolium bromide, Sigma‒Aldrich, Saint Louis, MO, USA) and incubated the plates for 2 h at 37 °C. After incubation, the MTT reagent was discarded, and 200 µL of acidified isopropanol (1 N HCl in 99.9% isopropanol) was added to dissolve the formazan crystals that had formed. Finally, we measured the absorbance at λ = 570 nm via a plate reader (FLUOstar Omega Ω) [11, 12].

### Western blotting

A 3 ml cell suspension was cultured in six-well cell culture plates supplemented with complete DMEM and then incubated at 37 °C in a 5% CO_2_ incubator for 24 h. After incubation, the supernatant was discarded, and the cell monolayer was washed with 3 ml of 1x PBS. The cell monolayer was then treated in duplicate according to the IC_50_. Duplicates for cell and virus control were included. The cell pellets were collected at 1, 3, 6, 12, and 24 h postinfection. The cell pellets were lysed with ice-cold RIPA lysis buffer supplemented with fresh protease inhibitor cocktail, incubated for 30 min on ice, and centrifuged at 15,000 RPM at 4 °C for 20 min, after which the supernatant was transferred to a fresh tube. The total water-soluble protein concentration was quantified via a bicinchoninic acid protein assay kit (Micro BCA™ Protein Assay Kit, REF: 23235; Thermo Scientific). Ten micrograms of protein were loaded in 2x Laemmli buffer and separated via 12% sodium dodecyl sulfate–polyacrylamide gel electrophoresis (SDS‒PAGE) along with a protein ladder (Cat. No. PM008-0500, GeneDirex, Taiwan). The proteins were transferred to a 0.2 μm PVDF membrane (REF.03010040001, Sigma Aldrich, St. Louis, MO, USA) by a Mini-PROTEAN^®^ Tetra Cell and Mini Trans-Blot (Bio-Rad) in transfer buffer (Bio-Rad Tris/Glycine transfer buffer Cat. #1610734) for 45 min at 100 V and 350 mA. β-actin (REF. No. MA5-11869; Invitrogen) was detected as a housekeeping protein against the Caspase-3 mAb (REF. No. 9662 S, Cell Signaling, Danvers, MA, USA) at a 1:1000 dilution via horseradish peroxidase-conjugated anti-mouse IgG (61-6520; Invitrogen) and anti-rabbit IgG (31460; Invitrogen) diluted 1:3000. Bands were detected via SuperSignal™ West Pico PLUS Chemiluminescent Substrate enhanced chemiluminescence (ECL, REF. 34577, Thermo Scientific, USA). Images were captured with a Bio-Rad ChemiDoc gel documentation imaging system and analyzed by Image Lab (Bio-Rad 6.1) [13].

### Molecular Docking

The crystal structure of the herpes simplex virus glycoprotein bound to the HVEM receptor (PDB ID: 1JMA) was retrieved from the RCSB Protein Data Bank [14]. To prepare the protein, it was imported into the PDB Reader and Manipulator module in CHARMMGUI. Initially, only chain A, which corresponds to the HVEM receptor, was retained for further processing. Missing hydrogen atoms were added, and the system’s pH was adjusted to 7.4 to ensure accurate protonation states of the residues. Additionally, residues 93 and 94 were modeled to address any gaps. The resulting. pdb file was then imported into Chimera, where partial charges were assigned via the AMBER ff14SB force field [15–18]. The modified protein structure was subsequently analyzed via fpocket and CASTp to identify potential binding sites [19]. CASTp identified a binding site involving residues Gln59, Cys81, Gly94, His96, and Cys97, which was ranked second, with a surface area of 15.855 Å² and a volume of 8.815 Å³. Similarly, the pocket al.so ranked second, with a pocket score of 24.7220, including residues Tyr45, Gln59, Thr76, Glu77, Asn78, Ala79, Val80, Cys81, Gly94, His96, and Cys97. Notably, the highest-ranked binding sites differed between the two methods, leading to the selection of this second-best site as the preferred binding site [20, 21].

The tested compounds (2, 3, and 5c) were drawn using ChemBioDraw Ultra. They were prepared by adding hydrogen atoms at pH 7, calculating partial charges via AM1-BCC, and performing energy minimization to the nearest local minimum via 1000 steps with the steepest descent algorithm in Chimera. The protein was then imported into GOLD software, where a cavity file was created to specify the residues comprising the binding site. The compounds were loaded into GOLD, generating 50 GA solutions for each compound, with ChemPLP as the scoring function. The resulting poses were clustered using a threshold of 1.5 Å with the complete linkage method. The best-ranked pose from the most populated cluster was saved as a ligand‒protein complex, which was then uploaded to the Protein‒Ligand Interaction Profiler (PLIP) and visualized via PyMOL [22–23].

## Results

### The cytotoxicity and antiviral activity of the compounds

**Fig. 3:**
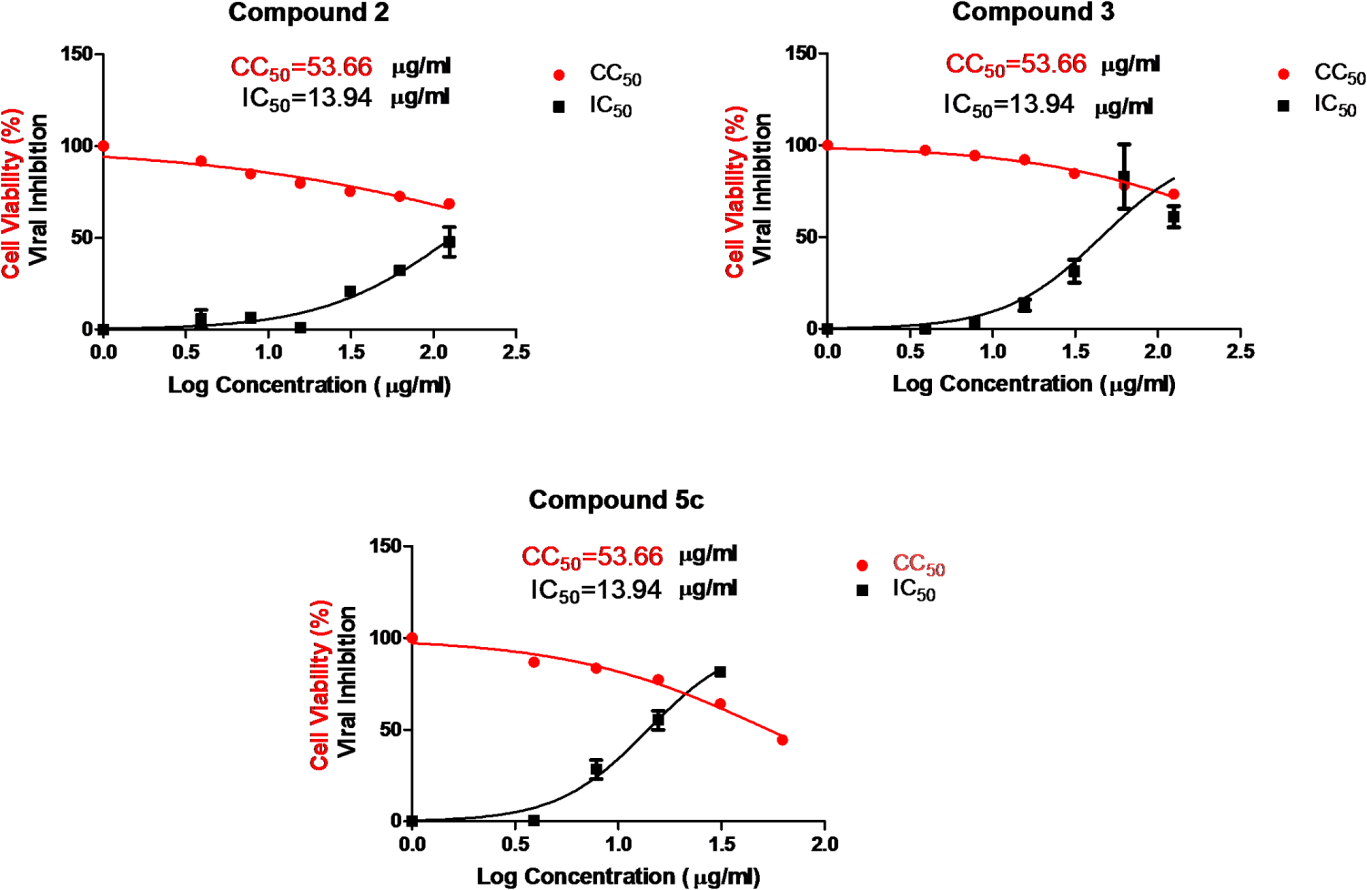
The plots of the CC_50_ and IC_50_ values are presented in Table 1 for the most promising compounds (2, 3, and 5c)

Recently, the synthesis of highly bioactive azo dye derivatives by the diazocoupling of aryl diazonium compounds with spirocyclic thiouracil and their antiviral activity against RNA viruses were evaluated [9, 24]. These compounds were prepared according to the authors’ previous protocol from the starting precursor 1 using three alkylating agents: methyl iodide, α-chloroacetylacetone, and α-chloroacetoacetanilide (Fig. 2). Two series of azo dye derivatives, 5a-f and 6a-c were generated through the diazotization of the two starting precursors, spirothiopyrimidinones 1 and 4, respectively, under basic conditions in satisfactory yields. Spiropyrimidinone 1 contains one active methine proton at the 5-position of the pyrimidinone nucleus, whereas the other derivative, 4, contains an additional active methine proton on the diketone fragment. The aryl diazonium salts were directed mainly toward the methine center of compound 4, which is in close proximity to the nitrile and carbonyl groups of the pyrimidinone nucleus. Various aryl diazonium salts have been developed utilizing aniline, *para*-substituted (chloro and methyl) aniline, and sulfa drugs as biological counterparts. The previous results of these compounds against RNA coronavirus encouraged the authors to investigate their antiviral activity against DNA viruses, particularly herpes simplex virus type 2. In this study, the authors evaluated the efficacy of 11 newly synthesized pyrimidine derivatives on the viability of Vero cells. The compounds were serially diluted and added to the cell culture, and the cell viability was determined after 72 h via crystal violet assay. The findings showed that most of the new derivatives were not toxic to Vero cells at doses up to 995 micrograms per milliliter (Table 1) Figure 3.

**Table 1:** Results of the cytotoxicity and inhibitory concentrations of the compounds in various cell lines

Compound	CC_50_ (µg/ml)	IC_50_ (µg/ml)	SI
2	565.5	130.1	4.34
3	509.30	45.42	11.21
5a	43.89	973.7	0.045
5b	124.3	536.3	0.231
5c	53.66	13.94	3.84
5d	465.5	217.1	2.14
5e	102.6	345.8	0.296
5f	107.0	398.6	0.268
6a	62.39	53.79	1.15
6b	940.1	5406	0.173
6c	42.09	748.8	0.056

### Inhibition mechanisms of action

**Fig. 4:**
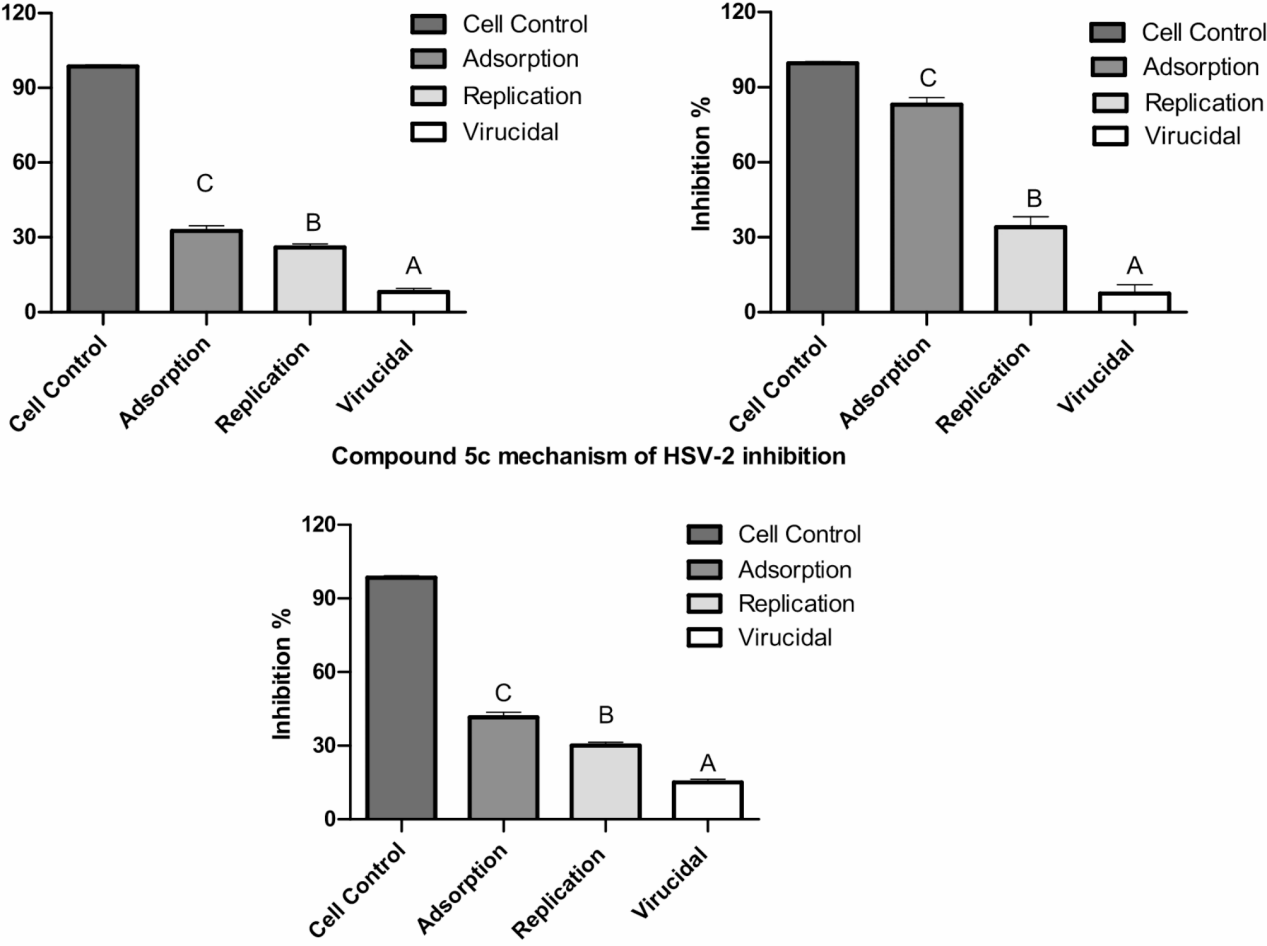
Mechanism of action of drug candidates 2, 3, and 5c in HSV-2 inhibition. The histograms show the different percentages of HSV-2 inhibition resulting from three different mechanisms of the viral infection life cycle: adsorption, replication, and virucidal. The histogram data are expressed as the mean ± S.D. of three independent experiments of viral inhibition mechanisms of action against cell control. Groups were statistically analyzed by f tests (ANOVAs) followed by post hoc Duncan tests. Groups with different letters are considered statistically significant at *p* < 0.05

**Fig. 5:**
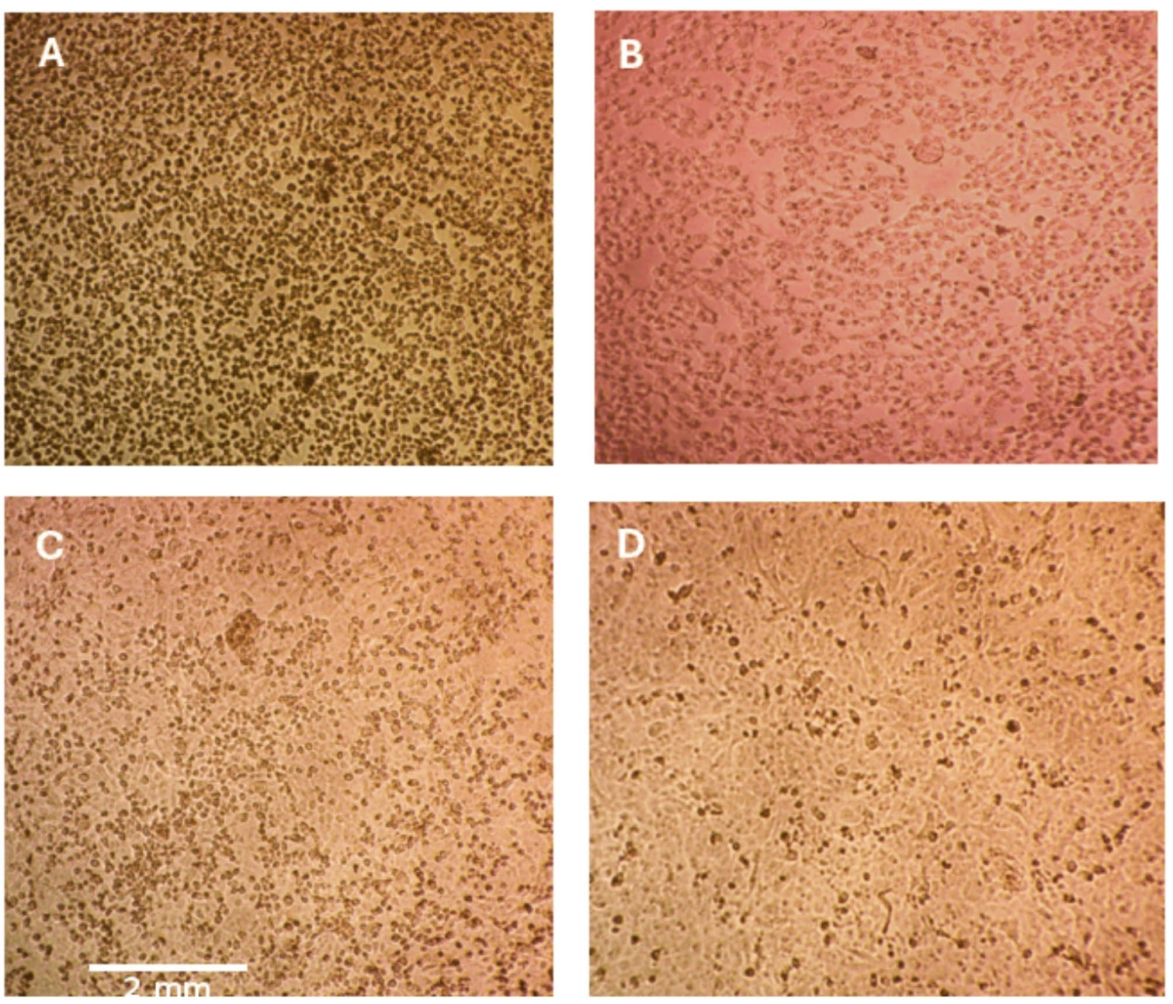
Microscopy images of the morphological changes in HSV-2-infected Vero cells (MOI = 1) treated with compound 3 via different viral infection mechanisms. **A**) Herpes virus control, **B**) infected Vero cells treated with 3 in the replication mechanism, **C**) infected Vero cells treated with 3 in the adsorption mechanism, and **D**) normal Vero cell control

The evaluation of antiviral activity revealed that compounds 2, 3, and 5c were the most promising new inhibitors of human herpes simplex virus type-2 (HSV-2) among all the tested compounds, which serve as antiviral models for DNA virus inhibition. These compounds demonstrated varying degrees of antiviral activity against HSV-2. The 50% inhibitory concentration (IC_50_) for these active compounds ranged from 13.9 to 130 µg/ml, as detailed in Table 1. Notably, compound 3 effectively suppressed HSV-2 with a favorable selectivity index of 11.2, indicating that it is a strong candidate for further development as an antiviral drug. This study also explored the mechanism of action of these three compounds to gain a deeper understanding of their inhibitory effects on HSV-2. These findings revealed that compound 3 had a significant effect on a crucial stage of the HSV-2 life cycle: viral adsorption. Specifically, 3 resulted in approximately 83% inhibition of HSV-2 adsorption, which is the process through which the virus attaches to and enters host cells. Furthermore, the results indicated that compound 3 also affected another important stage of the HSV-2 life cycle: viral replication, 34% inhibition, and 8% virucidal, where the drug directly affects the vial structure. Next, compound 5c had 42% inhibition of adsorption, 30% replication, and 15% virucidal mechanism. Finally, compound 2 inhibited adsorption by 32%, replication by 26%, and the virucidal mechanism by 8%. Importantly, the data showed that the compounds did not influence the virucidal process, indicating that they did not directly inactivate viral particles. (Fig. 4). Additionally, microscopy images revealed the effects of compound 3-induced HSV-2 inhibition through an adsorption mechanism on Vero cells compared with those of the control and control viruses. (Fig. 5)

### Western blot

**Fig. 6:**
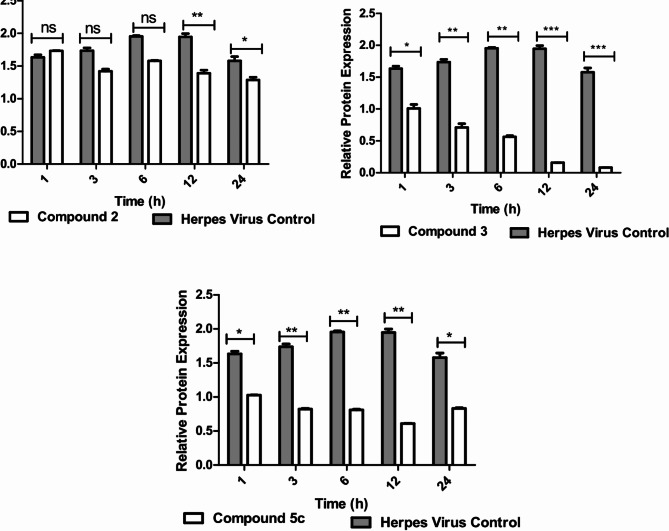
Western blot analysis for cleaved Caspase-3 protein expression in HSV-2-infected Vero cells (MOI = 1) treated with compounds 2, 3, and 5c compared with the HSV-2 virus control (infected Vero cells without any treatment (VC)). **A**) The protein expression level of β-actin at 1 h, 3 h, 6 h, 12 h, and 24 h postinfection. **B**) The protein expression level of Caspase-3 in infected HSV-2 virus control and infected Vero cells treated with 2, 3, or 5c. The measured band intensities of all the compound-treated cells and the virus control were plotted to compare the differences in the protein expression levels of Caspase-3. A *t*-test was used to compare the protein expression levels of caspase-3 in untreated and treated cells. ^*^p < 0.05, ^**^p < 0.01, and ^***^p < 0.005. However, ns is not significant

To further validate the impact of promising compounds in mitigating the cellular cytopathic effect (CPE) induced by viral infection, we examined the expression of the caspase-3 protein, which is crucial for the regulation of apoptosis. The objective was to determine whether the reduction in CPE observed with the tested compounds (2, 3, and 5c) was related to alterations in the apoptosis pathway. The results indicated that caspase-3 expression levels, as assessed by Western blotting, decreased at various postinfection time points (1, 3, 6, 12, and 24 h) in the drug-treated viral samples compared with those in the control samples. These findings suggest that the reduction in CPE caused by compound 3 may be specifically associated with its ability to HSV-2 viral infection, hence downregulating the expression of the caspase-3 protein. (Fig. 6).

### Molecular Docking

**Fig. 7:**
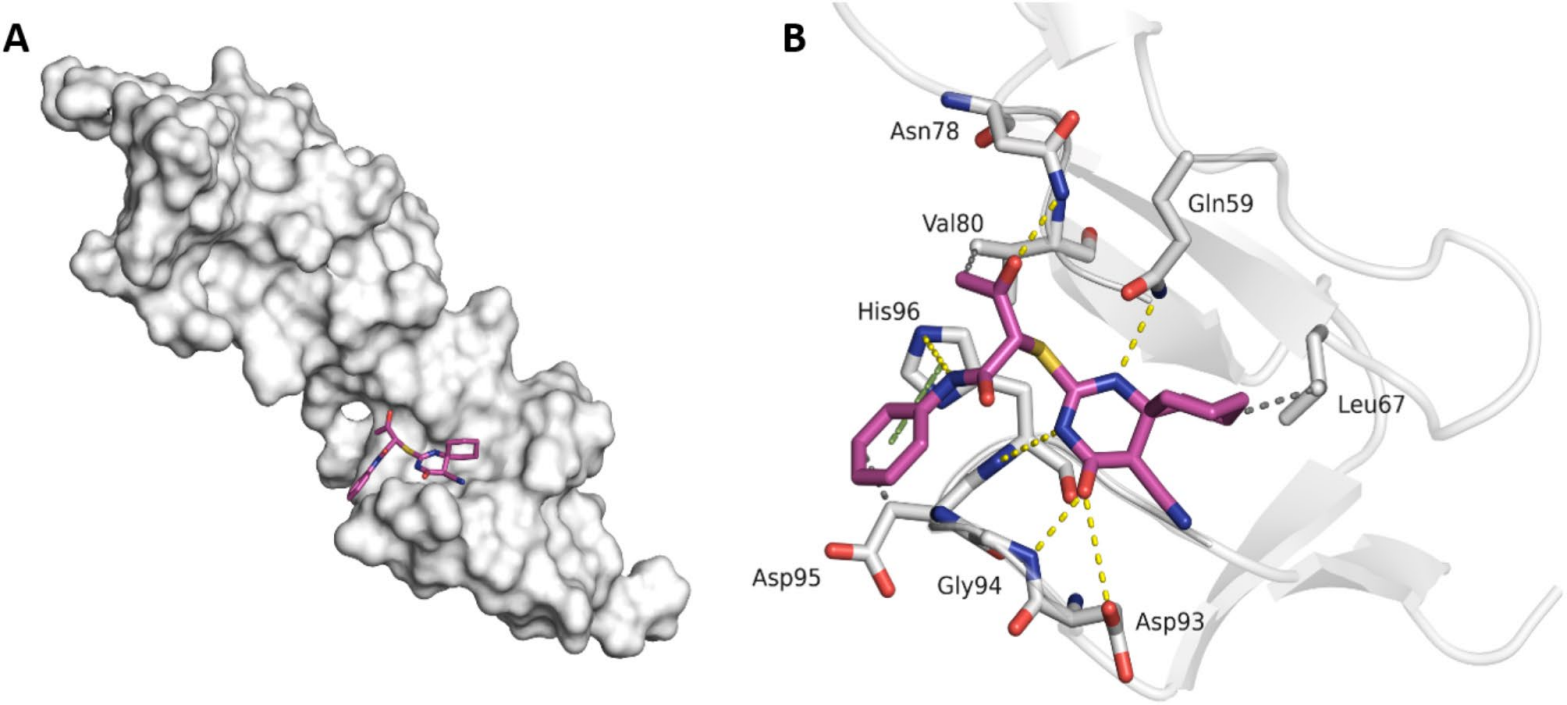
**A**) The best ranked binding mode of 3 in HVEM. **B**) The best ranked binding mode of 3 (magenta) in the binding pocket of HVEM (PDB ID 1JMA). Hydrogen bonding interactions, hydrophobic interactions and π‒π interactions are colored yellow, gray, and green, respectively

Finally, docking studies were conducted to investigate the binding mode of compound 3 to the herpes virus entry mediator (HVEM) (PDB ID: 1JMA). The diketone moiety is recognized for its role in enhancing the inhibitory effectiveness of related compounds against various viruses, including the NS2BNS3 proteases of the West Nile virus and the Mayaro virus [25, 26]. In our recent study [24], we reported that incorporating the PhNHOC-CH-COMe group into spiropyrimidine compounds led to a twofold increase in their antiviral activity against human coronavirus 229E (HCoV-229E). In the best-ranked binding mode of compound 3, several interactions were observed with the (PhNHOC-CH-COMe) group. Hydrogen bonds formed with the side chains of Asn78 and His96, whereas hydrophobic interactions were noted with Val80 via the terminal methyl group of compound 3 and with Asp95 through the phenyl moiety of the compound. Additionally, π‒π stacking interactions occurred between the phenyl group of compound 3 and the pyridine ring of His96 (Fig. 7).

## Discussion

Herpes simplex virus (HSV) is a human epidemic that poses a threat to public health. Globally, millions of people are exposed to herpes simplex virus (HSV), with a global seroprevalence of 66% [27], which includes latent infection in neurons, lytic infection of mucosal cells, and reactivation on a regular basis. The advanced immune evasion techniques of HSV lead to several illnesses, such as malignancy, newborn encephalitis, and genital lesions. An efficient preventive or therapeutic vaccination against HSV has not yet been developed. despite more than 70 years of continuous research, mainly because of our limited knowledge of virus‒host interactions. The human herpes simplex virus type-2 (HSV-2) is a member of the herpes viral family, which is a group of viruses that infect most people. It is the source of genital herpes, which is the most widespread sexually transmitted disease (STD) worldwide and causes mild to life-threatening infections in humans. It is often an undetected chronic infection that might increase the prevalence of human immunodeficiency virus (HIV). Antivirals are useful for limiting viral shedding and the duration of symptoms [28–30].

Hence, in this study, we demonstrate a chemical design that synthesizes a novel family of spirocyclic thiopyrimidinone derivatives by integrating several bioactive moieties (pyrimidine, cyano, azo, sulfonamide, diketone, and spirocycles) into a single hybrid. In this context, researchers have mentioned the antiviral effects of one or more members of the same chimeric family on low pathogenic corona virus (229E) [9, 31]. On the basis of these studies, we investigated the compounds’ cytotoxicity, antiviral efficacy, and mechanism of action of these new molecules against HSV-2.

At the cytotoxic level, the promising compounds presented various IC50 values ranging between 13.94 and 130 µg/ml. While the present data revealed that compound 3 presented the best SI of 11.21, with an IC_50_ of 45.42 µg/ml, compound 2 presented the best IC_50_ of 13.94 µg/ml, but its SI was only 4.3, indicating its toxicity to normal cells, with a CC_50_ of 565.5 µg/ml. Thus, compound 3 was selected for all downstream analyses, including an in-depth investigation of the mechanism of action to assess the suitable viral inhibition mechanism, whether it was replication, adsorption or virucidal.

Considering how the antiviral drugs affect HSV-2, compound 3 resulted in 83% viral inhibition by adsorption, 34% by replication, and only 8% by viricidal mechanisms when the host cells were compared with the normal control cells, indicating that adsorption is the major mechanism of viral inhibition for compound 3. While compound 5c showed 42% inhibition by adsorption, 30% inhibition by replication, and 15% inhibition by the virucidal mechanism, and compound 2 had 32% inhibition by adsorption and 26% inhibition by replication but only 8% inhibition by the virucidal mechanism. Among all the tested compounds, compound 3 is a promising candidate for further research [32].

The antiviral activity of compound 3 could be attributed to its diketone moiety (PhNHOC-CH-COMe), as the ketone group is well known for enhancing the inhibitory efficacy of related compounds against various viruses, including the NS2BNS3 protease of the West Nile virus and the Mayaro virus [26]. Furthermore, in our previous study [24], we reported that incorporating the PhNHOC-CH-COMe group into spiropyrimidine compounds resulted in a twofold increase in their antiviral activity against human coronavirus 229E (HCoV-229E).

In addition, the newly synthesized compounds resulted in varied levels of cleaved active form Caspase-3 protein expression, as determined by western blotting, compared with the level of expression in Herpes virus control cells. The western blot images revealed a significant decrease in caspase-3 protein levels at 1 h, 3 h, 6 h, 12 h, and 24 h post infection in the 3 treated groups. However, there were slight changes in the expression levels of these genes in the 2- and 5c-treated cells. Therefore, compound 3 inhibited HSV-2 viral infection, as reflected by the gradual downregulation of Caspase-3 protein expression at different time intervals postinfection [33].

In the docking studies of the spirothiopyrimidinone moiety of compound 3, its ketone group-maintained hydrogen bond interactions with the side chain of Asp93 and the backbone amide of Gly94. The nitrogen atoms of the pyrimidine ring also form hydrogen bonds with the side chain of Gln59 and the backbone of His96. Furthermore, hydrophobic interactions with Leu67 were observed. Consequently, the promising inhibitory effect of compound 3 against HSV-2 may be attributed to the interactions of both the spirothiopyrimidinone and (PhNHOC-CH-COMe) moieties within the binding pocket of HVEM, which differs from that of HSV-2. These findings suggest that compound 3 functions as an entry inhibitor by allosterically interacting with HVEM, altering its conformation and ultimately affecting HSV-2 virus adsorption [34].

Eventually, this study successfully identified compound 3 (2-((5-cyano-4-oxo-1,3-diazaspiro[5.5]undecan-2-yl)thio)-3-oxo-N-phenylbutanamide) as a potent inhibitor of HSV-2, contributing to the ongoing search for effective antiviral therapies against herpesvirus type 2 (HSV-2). These findings demonstrate that compound 3 interferes with critical stages of the HSV-2 life cycle, viral adsorption and replication while also exhibiting a favorable selectivity index and minimal cytotoxicity to normal cells. The association between the structure of 3, particularly its diketone moiety, and its antiviral activity underscores the potential for developing new antiviral agents on the basis of similar chemical frameworks. These docking studies provide insights into the binding interactions of compound 3 with HVEM, suggesting a novel mechanism of action as an entry inhibitor. Overall, these results pave the way for further investigation and optimization of this structural formula of compound 3 and its derivatives as viable therapeutic options for HSV-2 infections.

## Data Availability

No datasets were generated or analysed during the current study.

## Abbreviations

HSV-2: Herpes Simplex Virus type 2
HIV: Human Immunodeficiency Virus
STDs: Sexually transmitted diseases
CPE: Cytopathic effect
FBS: Fetal bovine serum
BSA: Bovine serum albumin
DMEM: Dulbecco's modified Eagle medium
PBS: Phosphate buffered sline
DMSO: Dimethyl sulfoxide
MTT: (3-(4, 5-dimethylthiazolyl-2)-2, 5-diphenyltetrazolium bromide) assay
CC50: Half maximal cytotoxcicity concentration
IC50: Half maximal inhibitory concentration
SI: Selective index
VC: Virus control
CC: Cell Control
SDS-PAGE: Sodium dodecyl-sulfate polyacrylamide gel electrophoresis
RIPA buffer: Radioimmunoprecipitation assay buffer
PVDF membrane: Polyvinylidene difluoride
HVEM: Herpes Virus Entry Mediator

## References

[1] Vaslin MFS, Arruda LB, Campos FS, Surveillance. Prevention, evolution and control of emerging viruses: A 2022 editorial update. Viruses. 2023;15(10):2098. 10.3390/v15102098. 37896875 10.3390/v15102098 PMC10612040

[2] Kausar S, Said Khan F, Ishaq Mujeeb Ur Rehman M, Akram M, Riaz M, Rasool G, Hamid Khan A, Saleem I, Shamim S, Malik A. A review: mechanism of action of antiviral drugs. Int J Immunopathol Pharmacol 2021 Jan-Dec;35:20587384211002621. 10.1177/20587384211002621 10.1177/20587384211002621 PMC7975490 33726557

[3] Spear PG, Longnecker R. Herpesvirus entry: an update. J Virol. 2003;77(19):10179–85. 10.1128/jvi.77.19.10179-10185.2003. 12970403 10.1128/JVI.77.19.10179-10185.2003 PMC228481

[4] https://www.who.int/news-room/fact-sheets/detail/herpes-simplex-virus

[5] Ostlund EN. The equine herpesviruses. Vet Clin North Am Equine Pract. 1993;9(2):283–94. 10.1016/s0749-0739(17)30396-6. 8395324 10.1016/s0749-0739(17)30396-6

[6] Omarova S, Cannon A, Weiss W, Bruccoleri A, Puccio J. Genital herpes simplex Virus-An updated review. Adv Pediatr. 2022;69(1):149–62. 10.1016/j.yapd.2022.03.010. 35985707 10.1016/j.yapd.2022.03.010

[7] Simmons A. Clinical manifestations and treatment considerations of herpes simplex virus infection. J Infect Dis. 2002;186(Suppl 1):S71–7. 10.1086/342967. 12353190 10.1086/342967

[8] Zhu S, Viejo-Borbolla A. Pathogenesis and virulence of herpes simplex virus. Virulence. 2021;12(1):2670–702. 10.1080/21505594.2021.1982373. 34676800 10.1080/21505594.2021.1982373 PMC8923070

[9] Ragab SS, Sweed AM, Hamza ZK, Shaban E, El-Sayed AA, Design. Synthesis, and antibacterial activity of spiropyrimidinone derivatives incorporated Azo sulfonamide chromophore for polyester printing application. Fib Polym. 2022;23:2114–22. 10.1007/s12221-022-4032-4.

[10] Allayeh AK, El-boghdady AH, Said MA, Saleh MGA, Abdel-Aal MT, Abouelenein MG. Discovery of Pyrano[2,3-c] pyrazole derivatives as novel potential human coronavirus inhibitors: design, synthesis, in Silico, in vitro, and ADME studies. Pharmaceuticals. 2024;17(198). 10.3390/ph17020198. 10.3390/ph17020198 PMC10892497 38399412

[11] Rasha Z, Batran; Ahmed S, Mohammed A, Khedr; Abdou K, Allayeh. Christophe Pannecouque; Asmaa F. Kassem; 4-Phenylcoumarin derivatives as new HIV-1 NNRTIs: design, synthesis, biological activities, and computational studies. Bioorg Chem. 2023;141:106918. 37866206 10.1016/j.bioorg.2023.106918

[12] Hend Hekal O, Hammad N, El-Brollosy M, Salem AK, Allayeh. Design, synthesis, docking, and antiviral evaluation of some novel pyrimidinone-based α-aminophosphonates as potent H1N1 and HCoV-229E inhibitors. Bioorg Chem. 2024;147:107353. 38615475 10.1016/j.bioorg.2024.107353

[13] Carfí A, Willis SH, Whitbeck JC, Krummenacher C, Cohen GH, Eisenberg RJ, Wiley DC. Herpes simplex virus glycoprotein D bound to the human receptor HveA. Mol Cell. 2001;8(1):169–79. 11511370 10.1016/s1097-2765(01)00298-2

[14] Jo S, Kim T, Iyer VG, Im W. CHARMM-GUI: A Web-based graphical user interface for CHARMM. J Comput Chem. 2008;29:1859–65. 18351591 10.1002/jcc.20945

[15] Jo S, Cheng X, Islam SM, Huang L, Rui H, Zhu A, Lee HS, Qi Y, Han W, Vanommeslaeghe K, MacKerell AD Jr., Roux B, Im W. CHARMM-GUI PDB manipulator for advanced modeling and simulations of proteins containing nonstandard residues. Adv Protein Chem Struct Biol. 2014;96:235–65. 25443960 10.1016/bs.apcsb.2014.06.002 PMC4739825

[16] S.J. Park, N.R. Kern, T. Brown, J. Lee, and W. Im (2023) CHARMM-GUI PDB Manipulator: Various PDB Structural Modifications for Biomolecular Modeling and Simulation (2023) J. Mol. Biol. 167995 10.1016/j.jmb.2023.167995 PMC10291205 37356910

[17] UCSF Chimera–a visualization system for exploratory research and analysis, Pettersen EF, Goddard TD, Huang CC, Couch GS, Greenblatt DM, Meng EC, Ferrin TE. J Comput Chem. 2004;25(13):1605-12. 10.1002/jcc.20084 15264254

[18] Guilloux VL, Schmidtke P, Tuffery P. Fpocket: an open-source platform for ligand pocket detection. BMC Bioinformatics. 2009;10:168. 19486540 10.1186/1471-2105-10-168 PMC2700099

[19] Tian et al. Nucleic Acids Res. 2018. PMID: 29860391 10.1093/nar/gky473

[20] Jones G, Willett P, Glen RC. Molecular recognition of receptor sites using a genetic algorithm with a description of desolvation. J Mol Biol. 1995;245(1):43–53. 10.1016/S0022-. 7823319 10.1016/s0022-2836(95)80037-9

[21] Jones G, Willett P, Glen RC, Leach AR, Taylor R. Development and validation of a genetic algorithm for flexible Docking. J Mol Biol. 1997;267(3):727–48. 10.1006/jmbi.1996.0897. 9126849 10.1006/jmbi.1996.0897

[22] Adasme MF, et al. PLIP 2021: expanding the scope of the protein–ligand interaction profiler to DNA and RNA. Nucl Acids Res (2 July. 2021;49(W1):W530–4. 10.1093/nar/gkab294. 10.1093/nar/gkab294 PMC8262720 33950214

[23] The PyMOL Molecular. Graphics System, Version 2.5.4 Schrödinger, LLC.

[24] Ragab SS, Sweed AMK, Elrashedy AA, Allayeh AK, Design. Synthesis, Antiviral Evaluation, and Molecular Dynamics Simulation Studies of New Spirocyclic Thiopyrimidinones as Anti HCoV-229E. Chem. Biodivers. 19 (2022) e202200632. Doi.10.1002/cbdv.202200632. 10.1002/cbdv.202200632 36097361

[25] Gazolla P, Oliveira A, Pereira W, Maia A, Santos E, Mendes T, Dias R, Polêto M, Teixeira R. Discovery of novel West nile virus protease inhibitor based on Isobenzonafuranone and Triazolic derivatives of Eugenol and indan-1,3-dione scaffolds. PLoS ONE. 2019;14:e0223017–0223017. 31557229 10.1371/journal.pone.0223017 PMC6762200

[26] Fernandes LS, da Silva ML, Dias RS, da S Lucindo MS, da Silva ÍE, Silva CC, Teixeira RR, de Paula SO. Evaluation of antiviral activity of Cyclic ketones against Mayaro virus. Viruses. 2021;13:2123–34. 34834929 10.3390/v13112123 PMC8625987

[27] Rebecca I, Montgomery ∙ Morgyn S, Warner ∙ Brian J. Lum ∙ Patricia G spear, herpes simplex Virus-1 entry into cells mediated by a novel member of the TNF/NGF receptor family. 87, Issue 3 P427–36 1996 Open Archive. 10.1016/s0092-8674(00)81363-x 8898196

[28] Poole CL, James SH. Antiviral therapies for herpesviruses: current agents and new directions. Clin Ther. 2018;40(8):1282–98. 10.1016/j.clinthera.2018.07.006. Epub 2018 Aug 10. PMID: 30104016; PMCID: PMC7728158. 30104016 10.1016/j.clinthera.2018.07.006 PMC7728158

[29] Helen O, Edogbanya SO, Momoh U, Sani. Herpes simplex virus type– 2 (HSV-2) and HIV coinfection dynamics with optimal control. Int J Dev Math 2024. 2024;1(1). 10.62054/ijdm/0101.09.

[30] Bai L, Xu J, Zeng L, et al. A review of HSV pathogenesis, vaccine development, and advanced applications. Mol Biomed. 2024;5:35. 10.1186/s43556-024-00199-7. 39207577 10.1186/s43556-024-00199-7 PMC11362470

[31] Ragab SS, Ibrahim NE, Abdel-Aziz MS, Elrashedy AA, Allayeh AK. Synthesis, biological activity, and molecular dynamic studies of new Triazolopyrimidine derivatives. Results Chem. 2023;6:101163. 10.1016/j.rechem.2023.101163.

[32] Lycke E, Johansson M, Svennerholm B, et al. Binding of herpes simplex virus to cellular Heparan sulphate, an initial step in the adsorption process. J Gen Virol. 1991;72(Pt 5):1131–7. 1851813 10.1099/0022-1317-72-5-1131

[33] Li L, Kovacs SB, Jørgensen I, Larson HN, Lazear HM, Miao EA. Role of caspases and gasdermin A during HSV-1 infection in mice. Viruses. 2022;14(9):2034. 10.3390/v14092034. 36146839 10.3390/v14092034 PMC9504851

[34] Madavaraju K, Koganti R, Volety I, Yadavalli T, Shukla D. Herpes simplex virus cell entry mechanisms: an update. Front Cell Infect Microbiol. 2021;10:617578. 10.3389/fcimb.2020.617578. 33537244 10.3389/fcimb.2020.617578 PMC7848091

